# The dietary inflammatory index and asthma prevalence: a cross-sectional analysis from NHANES

**DOI:** 10.3389/fnut.2024.1485399

**Published:** 2024-11-22

**Authors:** Chuansen Lu, Yike Zhu

**Affiliations:** ^1^Department of Neurology, Hainan General Hospital, Hainan Affiliated Hospital of Hainan Medical University, Haikou, China; ^2^Department of Respiratory and Critical Care Medicine, Hainan General Hospital, Hainan Affiliated Hospital of Hainan Medical University, Haikou, China

**Keywords:** asthma, dietary inflammatory index, NHANES, cross-sectional, inflammation

## Abstract

**Background:**

Inflammation is a key factor in the development of asthma, and diet significantly influences inflammatory responses. This study examines the relationship between the Dietary Inflammatory Index (DII) and asthma prevalence.

**Methods:**

We conducted a cross-sectional analysis using data from the National Health and Nutrition Examination Survey (NHANES) from 1999 to 2018. Demographic details, anthropometric measurements, dietary habits, lifestyle factors, and asthma status were recorded for all participants. Multivariable logistic regression was utilized to assess the relationship between DII and asthma prevalence. Additionally, restricted cubic spline (RCS) analysis was employed to explore the nonlinearity and dose–response relationship between DII and asthma risk. Subgroup analyses were stratified by gender, age, race, body mass index (BMI), poverty income ratio (PIR), education, smoking status, alcohol use, and family medical history to dissect the association between DII and asthma across diverse populations.

**Results:**

The analysis included 37,283 adults from NHANES. After adjusting for potential confounders in the multivariable logistic regression model, a significant positive association was identified between DII and asthma (OR, 95% CI: 1.05, 1.02–1.09, per 1 SD increase). The RCS analysis revealed a nonlinear association (*p* for nonlinearity = 0.0026), with an inflection point at 1.366, beyond which an increase in DII was significantly associated with asthma risk. Furthermore, the stratified analyses indicated a positive association between DII and asthma in the majority of subgroups.

**Conclusion:**

The findings underscore a significant and nonlinear association between DII and asthma. To enhance asthma prevention and management, greater emphasis should be placed on modulating dietary-induced inflammation.

## 1 Introduction

Asthma, a prevalent non-communicable disease, poses a significant threat to global health (1). The Global Burden of Disease Study estimated 262 million people to have asthma in 2019, reflecting an age-standardized prevalence of 3,416 cases per 100,000 individuals (2). While both genetic and non-genetic factors influence asthma (3), specific triggers are more likely than genetic factors to explain the rise in asthma prevalence among adults (4). Therefore, an improved comprehension of the adult-specific factors influencing asthma is essential for reducing the global burden of asthma.

Inflammation is widely recognized as a key factor in asthma development (5, 6). Prior studies have demonstrated a significant correlation between asthma and a spectrum of proinflammatory cytokines, encompassing interleukin-6 (IL-6), interleukin-33 (IL-33), interferon-gamma (IFN-*γ*), and tumor necrosis factor-alpha (TNF-*α*) (7–10). As an important modifiable exposure, diet has been demonstrated to have a significant influence on inflammatory processes. Dietary patterns characterized by high fiber, omega-3 polyunsaturated fatty acids, and green tea polyphenols have been correlated with decreased levels of inflammatory mediators (11–13). However, these nutrient-specific studies are limited by the fact that foods are consumed in complex combinations (14). Hence, the development of a comprehensive dietary index is essential for evaluating the diet-asthma association.

In 2014, Shivappa et al. (15) developed the Dietary Inflammatory Index (DII), designed to quantify the inflammatory potential of habitual diets. The DII is based on an extensive literature search incorporating cell culture, animal, and epidemiological studies on the effect of diet on inflammation. The DII encompasses not only micronutrients and macronutrients but also incorporates commonly consumed bioactive components including flavonoids, spices, and tea (16). Currently, an extensive body of research is exploring the links between DII and a variety of chronic noncommunicable diseases, such as obesity, type 2 diabetes, and cardiovascular diseases (17–19). Nonetheless, the relationship between asthma and DII remains unclear. Employing data from the National Health and Nutrition Examination Survey (NHANES), this study aims to explore the potential asthma-DII association.

## 2 Methods

### 2.1 Study population

The NHANES is an ongoing cross-sectional survey conducted once every 2 years by the Centers for Disease Control and Prevention of America (20). It is a research program designed to assess the health and nutrition status of residents in the United States. All participants in NHANES provided informed consent, and all protocols were approved by the Institutional Review Board of the Centers for Disease Control and Prevention (21). In the present study, all data were sourced from the NHANES conducted from 2003 to 2018. The exclusion criteria included: (1) age < 18 years, (2) participants without asthma data, and (3) participants without dietary data.

### 2.2 Dietary information

To minimize recall bias, dietary data were averaged from two 24-h recall interviews to calculate the DII score. The first dietary recall interview was collected in person in the Mobile Examination Center, and the second interview was collected by telephone 3–10 days later. The United States Department of Agriculture Food and Nutrient Database for Dietary Studies was used to calculate the nutrient intakes based on the foods and amounts reported in the survey (22). The DII, comprising 28 dietary components, was calculated for all subjects according to the protocol reported by Shivappa et al. (15). Briefly, six inflammatory markers (IL-1β, IL-6, IL-4, IL-10, TNF-*α*, and C-reactive protein) were used to evaluate the effect of the food parameter on inflammation (23). The “food parameter-specific overall inflammatory effect score” was calculated by study design and size of the literature for each food. All of the food parameter-specific scores were then summed to create the overall DII score for an individual. In the overall DII score, the positive value represents the pro-inflammatory potential of the diet, while the negative value represents the anti-inflammatory capacity.

### 2.3 Definition of asthma

Information regarding asthma was obtained from the health questionnaires. “Has a doctor or other health professional ever told you that you have asthma?” Participants who answered “yes” were regarded as asthma patients.

### 2.4 Assessment of other variables

The following data were collected: (1) demographics: age, sex, race, family income (poverty income ratio, PIR), and education levels were obtained from the demographic questionnaire; (2) body measurements: Body mass index (BMI) is defined as weight (kg)/height squared (m^2^); (3) lifestyle information: smoking and drinking status are collected using the health questionnaires; and (4) family history of asthma.

In this study, race was classified into Mexican American, Non-Hispanic White (NHW), Non-Hispanic Black (NHB), and others. Education was categorized into three levels: less than high school, high school or equivalent, and college or above. Based on Supplemental Nutrition Assistance Program eligibility, the PIR was divided into three levels: low-income level (PIR < 1.3), middle-income level (PIR 1.3–3.5), and high-income level (PIR > 3.5). According to the guidelines of the World Health Organization (WHO), a normal weight is defined as BMI < 25 kg/m^2^, overweight is defined as a BMI between 25 and less than 30 kg/m^2^ (25 to <30 kg/m^2^), and obesity is defined as BMI ≥  30 kg/m^2^.

### 2.5 Handling of missing variables

Data for all covariates were not complete, as shown in Supplementary Figure S1. To reduce bias from missing data, we performed multiple imputation to impute the missing data with all variables included in the analyses (Supplementary Figure S2). Missing values were imputed using chained equations with a 10-fold multiple imputation method.

### 2.6 Statistical analysis

Participants were classified into four groups based on quartiles of the DII score (from Quartile 1 to Quartile 4). Continuous variables are expressed as the mean ± standard deviation (SD), and categorical variables are expressed as percentages (95% confidence interval, 95% CI). To present differences in baseline characteristics between groups, *t*-tests and chi-Square tests were performed. The association between DII and asthma was explored with logistic regression models. Model 1 was unadjusted for any covariates. Model 2 included adjustments for age, gender, and race. Model 3 further adjusted for education, BMI, drinking, smoking, PIR, and family history in addition to the covariates in Model 2. To investigate the linearity and the dose–response relationship between DII and asthma, the restricted cubic spline (RCS) was performed with four knots placed at the 5th, 35th, 65th, and 95th percentiles. To evaluate heterogeneity among different populations, subgroup analyses stratified by sex, age, race, BMI, PIR, education, alcohol use, smoking status, and family history were conducted. All statistical analyses were performed with R software version 4.3.1 and *p* < 0.05 was considered statistically significant.

Finally, sensitivity analyses were conducted to validate the results. To eliminate the possible impact of missing data on the primary outcome, participants with any missing variable values were excluded.

## 3 Results

### 3.1 Baseline characteristics of participants

**Figure 1:**
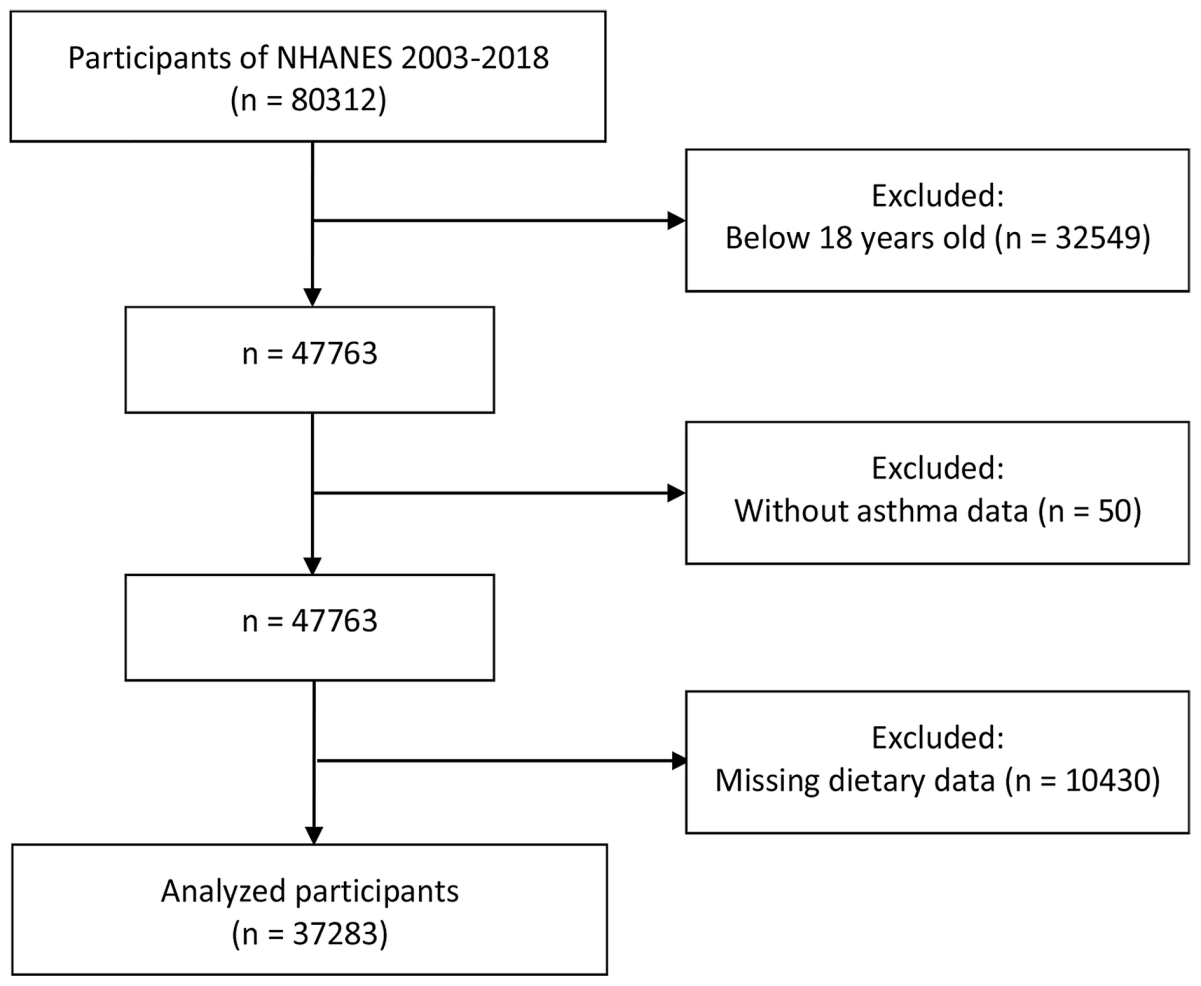
Flowchart of the study population.

A total of 80,312 participants were enrolled in the NHANES between 2003 and 2018. Among them, 32,549 individuals were excluded for being younger than 18 years. Participants without asthma data (*n* = 50) and those with missing dietary data (*n* = 10,430) were excluded from the remaining subjects. Finally, 37,283 participants were enrolled in our study, as shown in Figure 1.

Table 1 provides the baseline features of participants according to the quartiles of DII. The mean (SD) DII score for all participants was 1.186 (1.68). Compared with the lowest quartile group, subjects with higher DII scores were younger, more frequently female and NHB, had lower educational levels and household income, higher BMI, and a greater prevalence of family history of asthma. Moreover, individuals in higher DII quartiles reported higher rates of tobacco use and lower rates of alcohol consumption.

**Table 1:** Baseline characteristics of individuals classified by quartiles of the DII.

Characteristic	Overall	Quartiles of the DII				
		Quartile 1	Quartile 2	Quartile 3	Quartile 4	*p* value
Male, %	17,720 (47.5)	5,651 (60.6)	4,866 (52.2)	4,030 (43.2)	3,173 (34.0)	<0.001
Age, years	48.06 (18.97)	48.60 (18.14)	47.83 (18.55)	47.74 (19.28)	48.08 (19.87)	0.01
Race, %						<0.001
Mexican American	6,073 (16.3)	1,527 (16.4)	1,669 (17.9)	1,496 (16.1)	1,381 (14.8)	
Non-Hispanic Black	8,143 (21.8)	1,446 (15.5)	1799 (19.3)	2,342 (25.1)	2,556 (27.4)	
Non-Hispanic White	16,459 (44.1)	4,479 (48.0)	4,183 (44.9)	3,926 (42.1)	3,871 (41.5)	
Others	6,608 (17.7)	1870 (20.1)	1,669 (17.9)	1,556 (16.7)	1,513 (16.2)	
BMI, kg/m^2^	29.06 (6.95)	28.04 (6.28)	29.08 (6.85)	29.41 (7.12)	29.73 (7.41)	<0.001
Smoker, %						<0.001
Current	7,000 (19.7)	1,227 (13.6)	1,585 (17.7)	1838 (20.8)	2,350 (26.7)	
Past	8,842 (24.8)	2,555 (28.3)	2,351 (26.3)	2041 (23.1)	1895 (21.5)	
Never	19,770 (55.5)	5,255 (58.1)	4,997 (55.9)	4,967 (56.1)	4,551 (51.7)	
Drinker, %	24,713 (72.5)	6,749 (78.3)	6,383 (74.8)	6,036 (71.0)	5,545 (65.9)	<0.001
Education, %						<0.001
College or above	19,109 (51.3)	5,948 (63.8)	4,996 (53.7)	4,467 (48.0)	3,698 (39.7)	
High school	8,933 (24.0)	1784 (19.1)	2,173 (23.3)	2,376 (25.5)	2,600 (27.9)	
Less than high school	9,207 (24.7)	1,584 (17.0)	2,141 (23.0)	2,468 (26.5)	3,014 (32.4)	
PIR, %						<0.001
<1.3	10,785 (31.4)	2009 (23.2)	2,466 (28.7)	2,855 (33.2)	3,455 (40.4)	
1.3–3.5	13,123 (38.1)	3,066 (35.4)	3,259 (38.0)	3,435 (39.9)	3,363 (39.3)	
>3.5	10,493 (30.5)	3,574 (41.3)	2,856 (33.3)	2,321 (27.0)	1742 (20.4)	
Family history, %	7,558 (21.3)	1,674 (18.6)	1844 (20.7)	1960 (22.2)	2080 (23.6)	<0.001
DII	1.186 (1.68)	−1.11 (0.91)	0.75 (0.38)	1.92 (0.32)	3.18 (0.50)	<0.001

Continuous variables are expressed as the mean (standard deviation, SD). Categorical variables are expressed as proportion (95% confidence interval). PIR, Poverty income ratio; BMI, Body mass index; DII, Dietary inflammatory index.

In addition, the baseline characteristics of the study population grouped by asthma status are summarized in Supplementary Table S1. The distribution of the DII scores in this study is presented in Supplementary Figure S3.

### 3.2 Association between DII and asthma

There were 5,376 (14.4%) participants with asthma included in this analysis. The relationship between DII and asthma is detailed in Table 2. In Model 3, the odds ratios (ORs) across increasing quartiles of the DII were 1.00 (reference), 1.00 (95% CI 0.92–1.09), 1.03 (95% CI 0.94–1.12), and 1.16 (95% CI 1.06–1.27), respectively (*p* for trend < 0.001). Similarly, as a continuous variable, per SD unit increase in DII was associated with an increased risk of asthma (OR 1.05, 95%CI 1.02–1.09). After adjustment for covariates, a positive correlation between the DII and asthma was observed in Model 1 (OR, 95% CI: 1.08, 1.00–1.18, Q2; OR, 95% CI: 1.17, 1.08–1.28, Q3; OR, 95% CI: 1.40, 1.29–1.51, Q4; OR, 95% CI: 1.13, 1.10–1.17, per 1 SD increase) and Model 2 (OR, 95% CI: 1.06, 0.97–1.15, Q2; OR, 95% CI: 1.10, 1.01–1.20, Q3; OR, 95% CI: 1.27, 1.17–1.38, Q4; OR, 95% CI: 1.09, 1.06–1.13, per 1 SD increase).

**Table 2:** Association between the DII and asthma prevalence.

Variable	No. of Cases	No. of Participants	Odds Ratio (95% CI)		
			Model 1	Model 2	Model 3
DII					
Quartile 1	1,182	9,322	Reference	Reference	Reference
Quartile 2	1,267	9,320	1.08 (1.00,1.18)	1.06 (0.97,1.15)	1.00 (0.92,1.09)
Quartile 3	1,356	9,320	1.17 (1.08,1.28)	1.10 (1.01,1.20)	1.03 (0.94,1.12)
Quartile 4	1,571	9,321	1.40 (1.29,1.51)	1.27 (1.17,1.38)	1.16 (1.06,1.27)
*p* for trend			<0.001	<0.001	<0.001
Per 1 SD increase			1.13 (1.10,1.17)	1.09 (1.06,1.13)	1.05 (1.02,1.09)

Model 1, adjusted for none; Model 2, adjusted for age, gender, and race; Model 3, adjusted for adjusted for age, gender, race, education, BMI, drinking, smoking, PIR, and family history. DII, Dietary inflammatory index; OR, Odds ratio; CI, Confidence interval; SD, Standard deviation.

**Figure 2:**
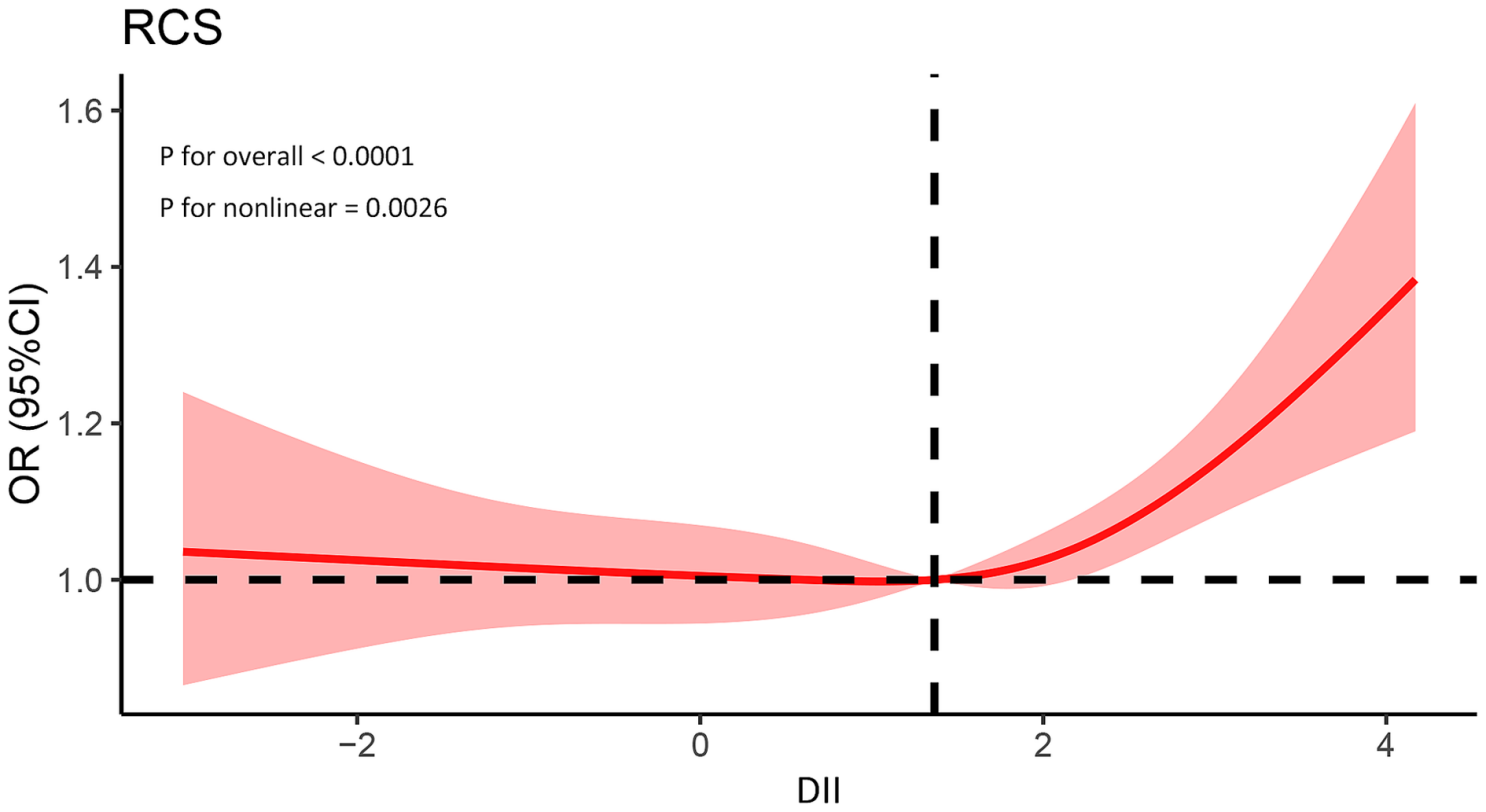
Association between the DII and prevalence of asthma. The model was adjusted for age, gender, race, education, BMI, drinking, smoking, PIR, and family history. PIR, Poverty income ratio; BMI, Body mass index; DII, Dietary inflammatory index.

In dose–response analyses, a significant nonlinear association between DII and asthma was observed in the fully adjusted RCS regression model (*p* for nonlinearity = 0.0026). Furthermore, we identified 1.366 as the inflection point. At this inflection point, the trajectory transitioned from flat to obliquely upward (Figure 2).

### 3.3 Subgroup analysis

**Figure 3:**
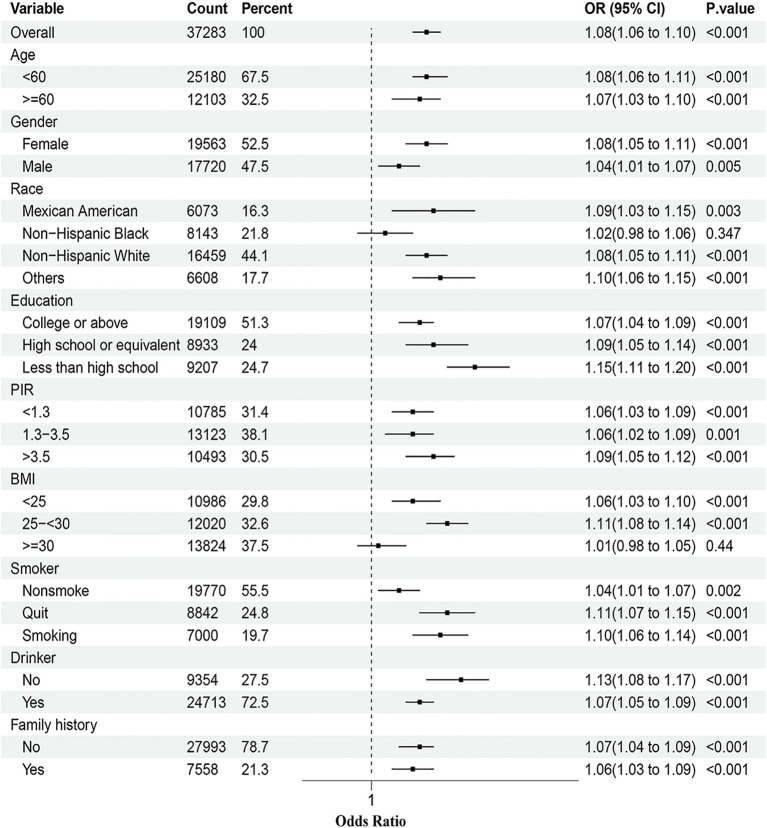
Subgroup analyses of the association between the DII and asthma prevalence. The model was adjusted for age, gender, race, education, BMI, drinking, smoking, PIR, and family history. PIR, Poverty income ratio; BMI, Body mass index; DII, Dietary inflammatory index.

To further explore the relationship between DII and asthma among different populations, a series of subgroup analyses were conducted. As shown in Figure 3, in the majority of strata, including age, gender, and education, the risk of asthma increases with the rise of the DII. However, this correlation was not significant in the subgroups of NHB and those with normal weight.

### 3.4 Sensitivity analysis

In sensitivity analyses, which aimed to eliminate the possible impact of missing values, the results were consistent with those of the main analysis. DII, whether as a continuous variable or a classification variable, exhibited a positive association with asthma in the sensitivity analysis (Supplementary Table S2).

## 4 Discussion

Studies on the relationship between DII and asthma are very limited and inconsistent. An Australian study of people aged over 12 years showed that asthmatics had a higher DII score than healthy controls (24). In another cross-sectional study, the findings did not support the hypothesis that a pro-inflammatory diet is associated with poorer asthma outcomes (25). However, these results were based on single studies with small sample sizes. To our knowledge, our study is the first with a large sample size to explore the relationship between DII and asthma among adults. In our study, a significant positive association between DII and asthma was observed. The association between the exposure and outcome variables remained even after adjusting for other covariates. Furthermore, the dose–response analysis displayed a nonlinear positive relationship, with an inflection point at 1.366. Stratified analyses revealed positive correlations between DII and asthma in the majority of subgroups.

Although existing studies on the role of the DII in asthma are still relatively rare, an increasing body of evidence supporting the protective effect of dietary antioxidants on asthma has emerged over the last decade. In a population-based study of Italian adults, high intakes of oleic acid and olive oil were associated with a lower risk of asthma (26). Another observational study, enrolling 2,506 participants from a Swedish birth cohort, demonstrated that higher intakes of fruits, vegetables, and dietary antioxidants were associated with reduced odds of asthma in school-age children (27). In addition, animal studies have shown that vitamin E can modulate allergic mediators and alleviate asthma symptoms while reducing pulmonary inflammation and airway mucus secretion in mice (28). The findings of our study further support these perspectives.

In recent years, a growing body of evidence indicates that diet may influence the onset and progression of asthma by modulating cellular oxidative stress responses and inflammatory mediator levels in the body (29, 30). According to a recent study, increased intakes of fruits and vegetables have been shown to reduce asthma-related illnesses and modulate cytokine production in peripheral blood mononuclear cells among young children with asthma (31). Additional research suggests that vitamin D inhibits airway smooth muscle cell contraction and remodeling, reduces inflammation, and may play a role in the prevention and management of asthma (32). In animal experiments, Lee et al. found that resveratrol, a natural polyphenol present in various fruits and vegetables, effectively ameliorated airway inflammation and structural changes in a murine model of bronchial asthma (33). These findings also provide a theoretical basis for further investigating the link between DII and asthma.

It is well established that gut microbiota plays a pivotal role in gut-lung axis regulation (34, 35). Diet serves as a significant modulator of gut microbial composition and associated metabolites (36, 37). Short-chain fatty acids, produced by the fermentation of dietary fiber by gut microbiota, can sculpt the immunological environment in the lungs and modulate the severity of allergic inflammation (38). Moreira et al. found that high-fat diets induce alterations in gut microbiota composition, enhance intestinal permeability, and trigger inflammation (39). In a cross-sectional study, a significantly higher abundance of Escherichia was observed in children with asthma compared to the healthy controls (40). The increased abundance of Escherichia is associated with a diet high in protein (41). These findings offer further insights, suggesting that diet may significantly influence the progression of asthma through diverse mechanisms.

The RCS is a set of smoothly connected piecewise polynomials that can clearly describe the relationship between independent and dependent variables (42). It was observed that when the DII exceeds 1.366, the risk of asthma is significantly elevated. These findings suggest that controlling the DII within a certain range is crucial. It may provide new insights for asthma prevention.

In subgroup analyses, the association between DII and asthma prevalence was consistent across most subgroups, which demonstrates the broad applicability of our findings to the majority of individuals. Available research has indicated that NHB have a significantly lower vegetable intake compared to NHW (43). Our results also revealed that NHB have higher DII scores, which could explain why the relationship between DII and asthma was not significant in the NHB subgroup. Furthermore, we observed that the correlation between DII and asthma prevalence was not significant in obese individuals. This may be attributed to the significant impact that adipose tissue has on asthma. Research indicates that an excess of adipose tissue may contribute to airway inflammation and exacerbate asthma symptoms (44).

A key strength of the current study is its large sample size, which provides reliable conclusions and robust statistical power. Additionally, the adoption of RCS analysis in our study further demonstrates the nonlinear associations between DII and asthma, potentially offering new evidence for health policymakers. Lastly, subgroup analyses and sensitivity analyses in this study enhanced the credibility of our assessment of the correlation between DII and asthma.

Some limitations of this study warrant clarification. Firstly, due to its cross-sectional design, this study can only suggest associations rather than infer causality. Furthermore, potential subjective bias may arise from self-reported dietary information and asthma status in the NHANES database. Finally, it should be noted that the current study was based on participants from the United States. Therefore, further research is essential to determine the universal applicability of these findings.

## 5 Conclusion

In conclusion, our study, based on NHANES data, indicated a nonlinear positive association between DII and asthma. This result suggests new avenues for reducing asthma prevalence rates through dietary interventions. Future prospective studies are urgently needed to confirm these findings and strengthen the basis for asthma prevention strategies.

## Data Availability

The datasets presented in this study can be found in online repositories. The names of the repository/repositories and accession number(s) can be found in the article/Supplementary material.

## References

[1] Porsbjerg C Melén E Lehtimäki L Shaw D. Asthma. Lancet. (2023) 401:858–73. doi: 10.1016/S0140-6736(22)02125-0 36682372

[2] GBD 2021 Diseases and Injuries Collaborators. Global incidence, prevalence, years lived with disability (YLDs), disability-adjusted life-years (DALYs), and healthy life expectancy (HALE) for 371 diseases and injuries in 204 countries and territories and 811 subnational locations, 1990–2021: A systematic analysis for the global burden of disease study 2021. Lancet. (2024) 403:2133–61. doi: 10.1016/S0140-6736(24)00757-8, PMID: 38642570 PMC11122111

[3] Stern J Pier J Litonjua AA. Asthma epidemiology and risk factors. Semin Immunopathol. (2020) 42:5–15. doi: 10.1007/s00281-020-00785-1 32020334

[4] Coumou H Westerhof GA de Nijs SB Amelink M Bel EH. New-onset asthma in adults: what does the trigger history tell us? J Allergy Clin Immunol Pract. (2019) 7:898–905.e1. doi: 10.1016/j.jaip.2018.09.007, PMID: 30240884

[5] Tattersall MC Jarjour NN Busse PJ. Systemic inflammation in asthma: what are the risks and impacts outside the airway? J Allergy Clin Immunol Pract. (2024) 12:849–62. doi: 10.1016/j.jaip.2024.02.004, PMID: 38355013 PMC11219096

[6] Banno A Reddy AT Lakshmi SP Reddy RC. Bidirectional interaction of airway epithelial remodeling and inflammation in asthma. Clin Sci. (2020) 134:1063–79. doi: 10.1042/CS20191309 32369100

[7] Peters MC Mauger D Ross KR Phillips B Gaston B Cardet JC. Evidence for exacerbation-prone asthma and predictive biomarkers of exacerbation frequency. Am J Respir Crit Care Med. (2020) 202:973–82. doi: 10.1164/rccm.201909-1813OC, PMID: 32479111 PMC7528796

[8] Saikumar Jayalatha AK Hesse L Ketelaar ME Koppelman GH Nawijn MC. The central role of IL-33/IL-1RL1 pathway in asthma: from pathogenesis to intervention. Pharmacol Ther. (2021) 225:107847. doi: 10.1016/j.pharmthera.2021.107847, PMID: 33819560

[9] Krusche J Twardziok M Rehbach K Böck A Tsang MS Schröder PC. TNF-α-induced protein 3 is a key player in childhood asthma development and environment-mediated protection. J Allergy Clin Immunol. (2019) 144:1684–1696.e12. doi: 10.1016/j.jaci.2019.07.029, PMID: 31381928

[10] Tiotiu A Badi Y Kermani NZ Sanak M Kolmert J Wheelock CE. Association of Differential Mast Cell Activation with granulocytic inflammation in severe asthma. Am J Respir Crit Care Med. (2022) 205:397–411. doi: 10.1164/rccm.202102-0355OC, PMID: 34813381

[11] Niero M Bartoli G De Colle P Scarcella M Zanetti M. Impact of dietary Fiber on inflammation and insulin resistance in older patients: a narrative review. Nutrients. (2023) 15:791–793. doi: 10.3390/nu15102365, PMID: 37242248 PMC10220584

[12] Borsini A Nicolaou A Camacho-Muñoz D Kendall AC Di Benedetto MG Giacobbe J. Omega-3 polyunsaturated fatty acids protect against inflammation through production of LOX and CYP450 lipid mediators: relevance for major depression and for human hippocampal neurogenesis. Mol Psychiatry. (2021) 26:6773–88. doi: 10.1038/s41380-021-01160-8, PMID: 34131267 PMC8760043

[13] Adamcakova J Balentova S Barosova R Hanusrichterova J Mikolka P Prso K. Effects of green tea polyphenol Epigallocatechin-3-Gallate on markers of inflammation and fibrosis in a rat model of pulmonary silicosis. Int J Mol Sci. (2023) 24: 797–799. doi: 10.3390/ijms24031857, PMID: 36768179 PMC9916388

[14] Hu FB. Dietary pattern analysis: a new direction in nutritional epidemiology. Curr Opin Lipidol. (2002) 13:3–9. doi: 10.1097/00041433-200202000-00002, PMID: 11790957

[15] Shivappa N Steck SE Hurley TG Hussey JR Hébert JR. Designing and developing a literature-derived, population-based dietary inflammatory index. Public Health Nutr. (2014) 17:1689–96. doi: 10.1017/S1368980013002115, PMID: 23941862 PMC3925198

[16] Hébert JR Shivappa N Wirth MD Hussey JR Hurley TG. Perspective: the dietary inflammatory index (DII)-lessons learned, improvements made, and future directions. Adv Nutr. (2019) 10:185–95. doi: 10.1093/advances/nmy071, PMID: 30615051 PMC6416047

[17] Wu L Shi Y Kong C Zhang J Chen S. Dietary inflammatory index and its association with the prevalence of coronary heart disease among 45, 306 US adults. Nutrients. (2022) 14:807–808. doi: 10.3390/nu14214553, PMID: 36364813 PMC9656485

[18] Denova-Gutiérrez E Muñoz-Aguirre P Shivappa N Hébert JR Tolentino-Mayo L Batis C. Dietary inflammatory index and type 2 diabetes mellitus in adults: the diabetes mellitus survey of Mexico City. Nutrients. (2018) 10:809–810. doi: 10.3390/nu10040385, PMID: 29561774 PMC5946170

[19] Zheng X Ge YZ Ruan GT Lin SQ Chen Y Liu CA. Association between the dietary inflammatory index and all-cause mortality in adults with obesity. Ann Nutr Metab. (2023) 79:434–47. doi: 10.1159/000533380, PMID: 37690445

[20] Wen J Wang C Giri M Guo S. Association between serum folate levels and blood eosinophil counts in American adults with asthma: results from NHANES 2011-2018. Front Immunol. (2023) 14:1134621. doi: 10.3389/fimmu.2023.1134621, PMID: 36911740 PMC9993087

[21] Centers for Disease Control and Prevention (2024). NHANES. Available online at: https://www.cdc.gov/nchs/nhanes/index.htm (Accessed October 30, 2024).

[22] Ahuja JK Moshfegh AJ Holden JM Harris E. USDA food and nutrient databases provide the infrastructure for food and nutrition research, policy, and practice. J Nutr. (2013) 143:241s–9s. doi: 10.3945/jn.112.170043, PMID: 23269654

[23] Phillips CM Chen LW Heude B Bernard JY Harvey NC Duijts L. Dietary inflammatory index and non-communicable disease risk: a narrative review. Nutrients. (2019) 11:820–822. doi: 10.3390/nu11081873, PMID: 31408965 PMC6722630

[24] Wood LG Shivappa N Berthon BS Gibson PG Hebert JR. Dietary inflammatory index is related to asthma risk, lung function and systemic inflammation in asthma. Clin Exp Allergy. (2015) 45:177–83. doi: 10.1111/cea.12323, PMID: 24708388 PMC4190104

[25] Visser E de Jong K van Zutphen T Kerstjens HAM Ten Brinke A. Dietary inflammatory index and clinical outcome measures in adults with moderate-to-severe asthma. J Allergy Clin Immunol Pract. (2023) 11:3680–3689.e7. doi: 10.1016/j.jaip.2023.08.032, PMID: 37652347

[26] Cazzoletti L Zanolin ME Spelta F Bono R Chamitava L Cerveri I. Dietary fats, olive oil and respiratory diseases in Italian adults: a population-based study. Clin Exp Allergy. (2019) 49:799–807. doi: 10.1111/cea.13352, PMID: 30689281

[27] Sdona E Ekström S Andersson N Hallberg J Rautiainen S Håkansson N. Fruit, vegetable and dietary antioxidant intake in school age, respiratory health up to young adulthood. Clin Exp Allergy. (2022) 52:104–14. doi: 10.1111/cea.14020, PMID: 34549838

[28] Jiang J Mehrabi Nasab E Athari SM Athari SS. Effects of vitamin E and selenium on allergic rhinitis and asthma pathophysiology. Respir Physiol Neurobiol. (2021) 286:103614. doi: 10.1016/j.resp.2020.103614, PMID: 33422684

[29] Alwarith J Kahleova H Crosby L Brooks A Brandon L Levin SM. The role of nutrition in asthma prevention and treatment. Nutr Rev. (2020) 78:928–38. doi: 10.1093/nutrit/nuaa005, PMID: 32167552 PMC7550896

[30] Garcia-Larsen V Del Giacco SR Moreira A Bonini M Charles D Reeves T. Asthma and dietary intake: an overview of systematic reviews. Allergy. (2016) 71:433–42. doi: 10.1111/all.12800, PMID: 26505989

[31] Hosseini B Berthon BS Jensen ME McLoughlin RF Wark PAB Nichol K. The effects of increasing fruit and vegetable intake in children with asthma on the modulation of innate immune responses. Nutrients. (2022) 14:863–864. doi: 10.3390/nu14153087, PMID: 35956264 PMC9370535

[32] Salameh L Mahmood W Hamoudi R Almazrouei K Lochanan M Seyhoglu S. The role of vitamin D supplementation on airway remodeling in asthma: a systematic review. Nutrients. (2023) 15:866–867. doi: 10.3390/nu15112477, PMID: 37299440 PMC10255490

[33] Lee HY Kim IK Yoon HK Kwon SS Rhee CK Lee SY. Inhibitory effects of resveratrol on airway remodeling by transforming growth factor-β/Smad signaling pathway in chronic asthma model. Allergy, Asthma Immunol Res. (2017) 9:25–34. doi: 10.4168/aair.2017.9.1.25, PMID: 27826959 PMC5102832

[34] Song XL Liang J Lin SZ Xie YW Ke CH Ao D. Gut-lung axis and asthma: a historical review on mechanism and future perspective. Clin Transl Allergy. (2024) 14:e12356. doi: 10.1002/clt2.12356, PMID: 38687096 PMC11060082

[35] Samuelson DR Welsh DA Shellito JE. Regulation of lung immunity and host defense by the intestinal microbiota. Front Microbiol. (2015) 6:1085. doi: 10.3389/fmicb.2015.01085, PMID: 26500629 PMC4595839

[36] Zmora N Suez J Elinav E. You are what you eat: diet, health and the gut microbiota. Nat Rev Gastroenterol Hepatol. (2019) 16:35–56. doi: 10.1038/s41575-018-0061-2, PMID: 30262901

[37] Beam A Clinger E Hao L. Effect of diet and dietary components on the composition of the gut microbiota. Nutrients. (2021) 13:877–878. doi: 10.3390/nu13082795, PMID: 34444955 PMC8398149

[38] Trompette A Gollwitzer ES Yadava K Sichelstiel AK Sprenger N Ngom-Bru C. Gut microbiota metabolism of dietary fiber influences allergic airway disease and hematopoiesis. Nat Med. (2014) 20:159–66. doi: 10.1038/nm.3444, PMID: 24390308

[39] Moreira AP Texeira TF Ferreira AB Peluzio Mdo C Alfenas RC. Influence of a high-fat diet on gut microbiota, intestinal permeability and metabolic endotoxaemia. Br J Nutr. (2012) 108:801–9. doi: 10.1017/S0007114512001213, PMID: 22717075

[40] Chiu CY Chan YL Tsai MH Wang CJ Chiang MH Chiu CC. Gut microbial dysbiosis is associated with allergen-specific IgE responses in young children with airway allergies. World Allergy Organ J. (2019) 12:100021. doi: 10.1016/j.waojou.2019.100021, PMID: 30937143 PMC6439417

[41] Beaumont M Portune KJ Steuer N Lan A Cerrudo V Audebert M. Quantity and source of dietary protein influence metabolite production by gut microbiota and rectal mucosa gene expression: a randomized, parallel, double-blind trial in overweight humans. Am J Clin Nutr. (2017) 106:1005–19. doi: 10.3945/ajcn.117.158816, PMID: 28903954

[42] Núñez E Steyerberg EW Núñez J. Regression modeling strategies. Rev Esp Cardiol. (2011) 64:501–7. doi: 10.1016/j.recesp.2011.01.019, PMID: 21531065

[43] Thompson TL Singleton CR Springfield SE Thorpe RJ Jr Odoms-Young A. Differences in nutrient intake and diet quality between Non-Hispanic Black and Non-Hispanic White men in the United States. Public Health Rep. (2020) 135:334–42. doi: 10.1177/0033354920913058 32250708 PMC7238700

[44] Pérez-Pérez A Vilariño-García T Fernández-Riejos P Martín-González J Segura-Egea JJ Sánchez-Margalet V. Role of leptin as a link between metabolism and the immune system. Cytokine Growth Factor Rev. (2017) 35:71–84. doi: 10.1016/j.cytogfr.2017.03.001, PMID: 28285098

