# Supplementary figures and images for: The dietary inflammatory index and asthma prevalence: a cross-sectional analysis from NHANES
Source: Front Nutr. 2024 Nov 22;11:1485399. doi: 10.3389/fnut.2024.1485399 (PMC11622817; doi:10.3389/fnut.2024.1485399)

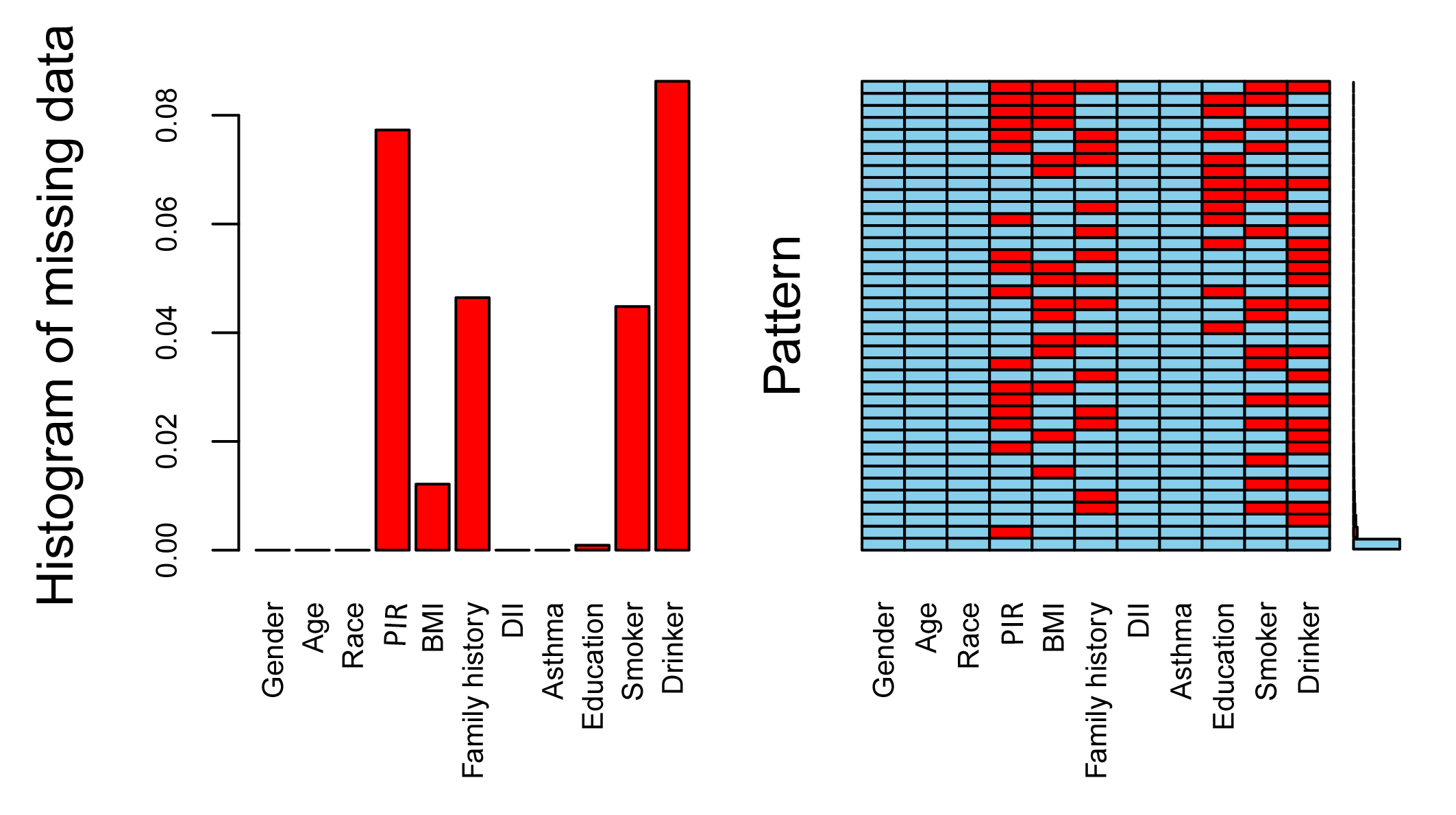

Supplement: Supplementary file 1 [file Image_1.TIF]

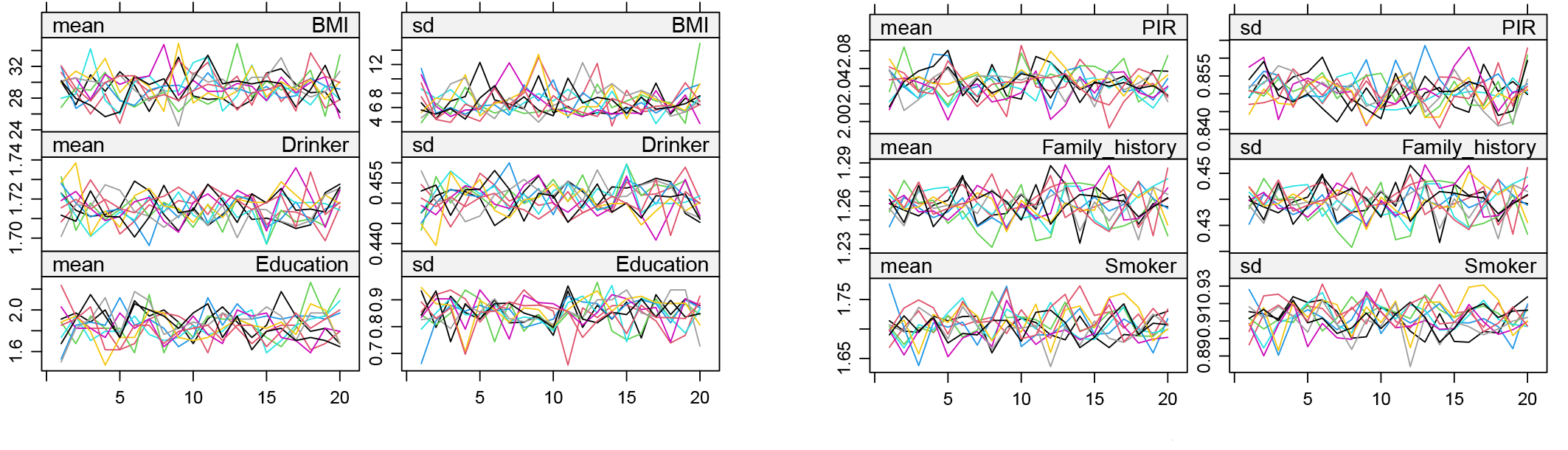

Supplement: Supplementary file 2 [file Image_2.TIF]

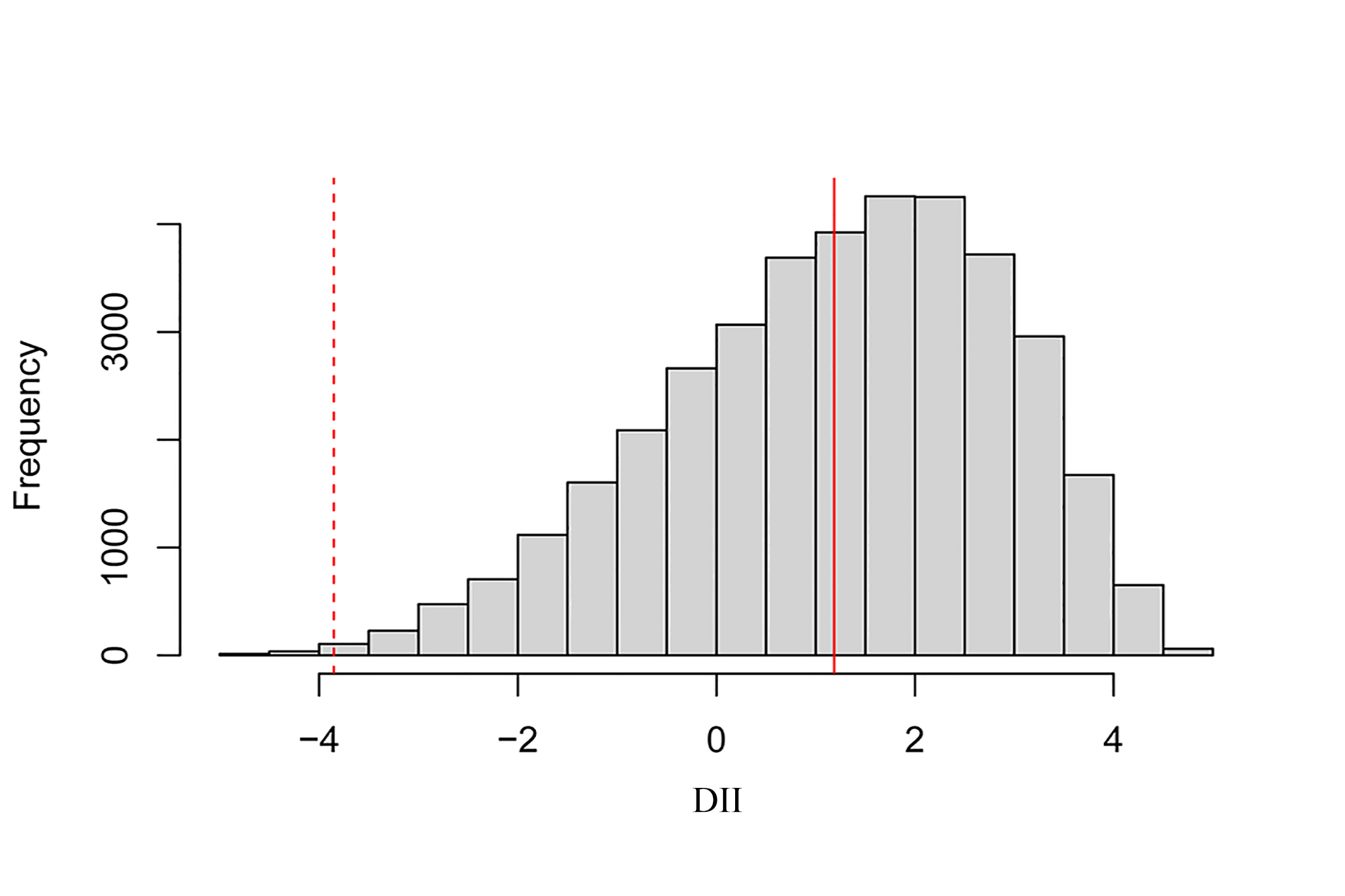

Supplement: Supplementary file 3 [file Image_3.TIF]
