# Supplementary material for: The dietary inflammatory index and asthma prevalence: a cross-sectional analysis from NHANES
Source: Front Nutr. 2024 Nov 22;11:1485399. doi: 10.3389/fnut.2024.1485399 (PMC11622817; doi:10.3389/fnut.2024.1485399)
Supplement: Supplementary file 4 [file Table_1.DOCX]

| Characteristic | Overall | No asthma | With asthma | P-value |
| --- | --- | --- | --- | --- |
|  | (n=37283) | (n=31907) | (n=5376) |  |
| Male,% | 17720 (47.5) | 15497 (48.6) | 2223 (41.4) | <0.001 |
| Age,years | 48.06(18.97) | 48.48 (18.97) | 45.57 (18.79) | <0.001 |
| Race,% |  |  |  | <0.001 |
| Mexican American | 6073(16.3) | 5533 (17.3) | 540 (10.0) |  |
| Non-Hispanic black | 8143(21.8) | 6780 (21.2) | 1363 (25.4) |  |
| Non-Hispanic white | 16459(44.1) | 13924 (43.6) | 2535 (47.2) |  |
| Others | 6608(17.7) | 5670 (17.8) | 938 (17.4) |  |
| BMI,kg/m2 | 29.06(6.95) | 28.81 (6.70) | 30.59 (8.15) | <0.001 |
| Smoker,% |  |  |  | <0.001 |
| Current | 7000(19.7) | 17184 (56.3) | 2586 (50.8) |  |
| Past | 8842(24.8) | 7548 (24.7) | 1294 (25.4) |  |
| Never | 19770(55.5) | 17184 (56.3) | 2586 (50.8) |  |
| Drinker,% | 24713(72.5) | 21064 (72.3) | 3649 (74.0) | 0.011 |
| Education,% |  |  |  | <0.001 |
| College or above | 19109(51.3) | 16201 (50.8) | 2908 (54.1) |  |
| High school or equivalent | 8933(24.0) | 7678 (24.1) | 1255 (23.4) |  |
| Less than high school | 9207(24.7) | 7998 (25.1) | 1209 (22.5) |  |
| Pir,% |  |  |  | <0.001 |
| <1.3 | 10785(31.4) | 8932 (30.4) | 1853 (37.2) |  |
| 1.3-3.5 | 13123(38.1) | 11392 (38.7) | 1731 (34.7) |  |
| >3.5 | 10493(30.5) | 9092 (30.9) | 1401 (28.1) |  |
| Family history,% | 7558(21.3) | 5390 (17.7) | 2168 (43.0) | <0.001 |
| DII | 1.186(1.68) | 1.16 (1.66) | 1.36 (1.66) | <0.001 |

Table S1: Baseline characteristics of individuals classified by outcome.
