# Supplementary material for: The dietary inflammatory index and asthma prevalence: a cross-sectional analysis from NHANES
Source: Front Nutr. 2024 Nov 22;11:1485399. doi: 10.3389/fnut.2024.1485399 (PMC11622817; doi:10.3389/fnut.2024.1485399)
Supplement: Supplementary file 5 [file Table_2.docx]

Table S2: Association between the DII and asthma prevalence after excluding individuals with any missing value

| Variable | No. of Cases | No. of Participants | Odds Ratio (95% CI) | | |
| --- | --- | --- | --- | --- | --- |
|  |  |  | Model 1 | Model 2 | Model 3 |
| DII |  |  |  |  |  |
| Quartile 1 | 897 | 7238 | Reference | Reference | Reference |
| Quartile 2 | 996 | 7237 | 1.13(1.02,1.24) | 1.09(0.99,1.21) | 1.04(0.94,1.15) |
| Quartile 3 | 1014 | 7238 | 1.15(1.05,1.27) | 1.07(0.97,1.18) | 1.01(0.91,1.11) |
| Quartile 4 | 1225 | 7238 | 1.44(1.31,1.58) | 1.30(1.18,1.43) | 1.18(1.07,1.31) |
| P for trend |  |  | <0.001 | <0.001 | 0.003 |
| Per 1 SD increase |  |  | 1.13(1.10,1.17) | 1.09(1.05,1.13) | 1.05(1.01,1.09) |
